# Frequency of Acute Kidney Injury in tetanus patients of Paedriatic Intensive Care Unit: A Public Hospital Experience

**DOI:** 10.12669/pjms.342.14254

**Published:** 2018

**Authors:** Faizia Naseem, Abid Hussain, Fehmina Arif

**Affiliations:** 1 Faizia Naseem, MBBS, DCH, MCPS, FCPS. Assistant Professor Department of Paediatrics, Dow Medical College, Dow University of Health Sciences, Karachi, Pakistan; 2 Abid Hussain, MBBS, DCH, FCPS. Assistant Professor Department of Paediatrics, Dow Medical College, Dow University of Health Sciences, Karachi, Pakistan; 3 Fehmina Arif, MBBS, DCH, FCPS. Professor, Department of Paediatrics, Dow Medical College, Dow University of Health Sciences, Karachi, Pakistan

**Keywords:** Tetanus, Acute Kidney injury (AKI)

## Abstract

**Background and Objective:**

Tetanus is a potentially fatal but preventable disease. Mortality is related to severity of the disease, cardiovascular, pulmonary and renal complications. Acute kidney injury (AKI) is a frequent and lethal complication of tetanus. The objective was to determine the frequency of AKI in tetanus patients managed in a public hospital.

**Methods:**

Children aged 1-12 years admitted in Paediatric Intensive Care Unit (PICU) with the clinical diagnosis of tetanus over three and half years were recruited for the retrospective study. pRIFLE (Pediatric Risk, Injury, Failure, Loss, End) criteria was applied to all cases of tetanus to categorize them as having AKI or not, on the basis of estimated creatinine clearance (ECCL). Comparison was done between AKI and non-AKI cases, as well as between AKI survivors and AKI non-survivors. The study was conducted at PICU of Dr. Ruth K.M. PFau Civil Hospital Karachi for tetanus cases admitted during July 2013 to December 2016

**Results:**

During the study period, 44 patients of tetanus were enrolled. Nearly 32 % of tetanus patients developed acute renal dysfunction according to PRIFLE criteria. There were overall 15 (34.09%) expiries among tetanus patients among which nine (60%) had AKI. Oliguria was observed in five (35.71%) cases. All the AKI non-survivors had ECCL below 50% and all had autonomic instability. AKI developed towards the end of first week in three cases, mid of second week in four cases and third week in seven cases. Renal replacement therapy (RRT) i.e. peritoneal dialysis (PD) was done in four AKI cases but it did not improve the outcome. CRP was more than 50 in 24 (54.54%) cases. Ventilatory support was given to 85.71% with AKI as compared to 66.66% of non AKI patients.

**Conclusion:**

Development of AKI in tetanus is multifactorial. Major contributors are severity of the tetanus itself, presence of autonomic instability, ventilator dependency, and sepsis. Presence of AKI worsens the outcome of tetanus in terms of survival, length of stay, hospital cost and ventilator days.

## INTRODUCTION

Among hundreds of infectious diseases known today, tetanus is a potentially fatal disease, which still remains an important public health problem in developing countries^1^, which requires continuous and strong efforts of both public and private sectors for the eradication. The current burden of tetanus in Pakistan is alarming like other vaccine preventable diseases although the exact figures are not available. Tetanus is caused by clostridium tetani, which produces a powerful neurotoxin, tetanospasmin affecting the central nervous system.^2^ Patient develops frequent body spasms, locked jaw and dysphagia.^3^ Wide swings in blood pressure and heart rate are common secondary to autonomic instability due to disinhibited autonomic discharge.^4^-^6^ Neuronal binding of toxin is irreversible and clinical recovery follows the growth of new nerve terminals explaining the usual prolonged course of illness.^7^ Treatment is aimed at neutralizing the toxin, controlling the muscle spasms, stabilizing the autonomic instability, managing the wound and monitoring the vital organ’s functioning. Multiple drugs are given simultaneously to achieve these goals.

Various factors have been related to high mortality rates seen in tetanus like grade of severity of the disease itself and development of cardiovascular, pulmonary and renal complications.^8^

Acute kidney injury (AKI) is a frequent and lethal complication of tetanus.^9^ A recent multivariate analysis of 236 ICU tetanus patients disclosed a direct association between AKI and tetanus mortality.^10^ This study was carried out to find the frequency of AKI in tetanus patients managed in a public hospital in Karachi, (to prevent AKI in future, in such cases).

## METHODS

This was a retrospective study. Children aged one month to twelve years admitted in PICU with the clinical diagnosis of tetanus over past three and half years i.e. from July 2013 to December 2016 were recruited for the study. Data were collected and reviewed form the file records for the course of illness, development of AKI, length of stay in PICU and the final outcome. As per PICU protocol, all the admitted tetanus patients were monitored for fluid volume and perfusion status, blood pressure centiles, input and output. Blood biochemistry, renal function tests and urine analysis was done in all patients.

### Inclusion Criteria

Children aged one month to twelve years with clinical diagnosis of tetanus without pre-existing kidney disease.

### Exclusion Criteria

Children with tetanus having preexisting kidney disease.

One case was excluded from the review on this basis with abnormal renal functions and ultrasound scan.

pRIFLE criteria was applied to all cases of tetanus to categorize them as having AKI or not, on the basis of Estimated Creatinine Clearance (ECCL). It was calculated on daily basis and the drug dosage adjustments were done accordingly. All the patients were also monitored for signs of autonomic instability, seen commonly in tetanus.

Data were analyzed using SPSS version 16. Descriptive statistics were applied to describe the results in terms of frequencies and percentages. Comparison was done between AKI and non-AKI cases, as well as between AKI survivors and AKI non-survivors. P values < 0.05 was considered as significant.

## RESULTS

During the study period, 44 patients of tetanus were enrolled (see Table-I for descriptive characteristics). Fourteen (31.81%) tetanus patients developed acute renal dysfunction according to PRIFLE criteria. There were overall 15 (34.09%) expiries among tetanus patients out of which 9 (60%) had AKI. (Table-II)

**Table-I:** Descriptive characteristics of tetanus patients (Total No. = 44.).

*Characteristic*	No	%
1.Sex:		
Male	24	54.54
Female	20	45.45
2.Age groups:		
< than 5 year	22	50
6 – 9 year	14	31.81
> 10 year	08	18.18
3.Grade of Severity:		
Grade I	0	0
Grade II	7	15.90
Grade III	27	61.36
Grade IV	10	22.72
4.Developed AKI:		
Yes	14	31.81
No	30	68.18
5.Final Outcome:		
Discharge	29	65.90
Expiry	15	34.09
6.Autonomic Instability	37	84.09
7.Ventilator requirement	32	72.72
8.CRP:		
< 50	20	45.45
> 50	24	54.54
9.TLC:		
<5,000/mm^3^	5	11.36
>20,000/mm^3^	39	88.63
10.Average length of stay (days):		
20 days	30	68.18
25 days	14	31.81

**Table-II:** Characteristics of tetanus patients with AKI (AKI +VE) Vs without AKI (AKI -VE) (Total No. = 44).

Characteristic	AKI +ve (14)		AKI -ve (30)		P- Value

	No	%	No	%	%
1.Expiries	9	64.28	6	20	0.004
2.Blood Culture Positively	6	66.66	9	30	0.001
3.TLC >20,000/mm^3^	11	78.57	6	20	0.001
4.CRP >50	10	71.42	14	46.66	0.56
5.Ventilator requirement	12	85.71	20	66.66	0.188
6.Severity of tetanus:					
Grade III	10	71.42	17	56.66	0.52
Grade IV	4	28.57	6	20.0	
7.Average length of stay (days)	25	-	20	-	-

Among the AKI cases, none belonged to grade I or II tetanus severity i.e. all were severe and very severe cases according to ‘Ablett classification’ of tetanus severity. Oliguria was observed in five (35.71%) cases. Urine analysis was insignificant in most cases; mild proteinuria was observed in four cases. Urine was not checked for myoglobinuria.

AKI developed towards the end of first week in three cases, mid of second week in four cases and third week in seven cases. Since the AKI was categorized according to pRIFLE criteria, four patients had estimated creatinine clearance between 75 to 50% and 10 patients had ‘ECCL’ below 50% (Table-II). All the AKI non-survivors had ECCL below 50% (Table-III).

**Table-III:** AKI Survivors Vs AKI Non-Survivors (Total AKI victims = 14).

Characteristics	Survivors	Non-Survivors	P. Value
1.Number	5	9	0.004
2.Grade of Severity:			
Grade III	5	5	0.001
Grade IV	0	4	
3.Ventilator requirement	3	9	0.135
^4.^TLC: >20,000/mm^3^	3	8	0.012
5.Blood Culture positively	1	5	0.001
6.CRP > 100	1	6	0.098
7.Creatinine clearance b/w:			
75 – 50	4	0	0.206
< 50	1	9	

All those who developed AKI had autonomic instability with episodes of tachycardia, bradycardia, hypertension, hypotension and all of them required inotropes to sustain their heart rate and blood pressure norms. Ventilator requirement was seen in 12 out of 14 AKI victims (85.71%), whereas 20 out of 30 (66.66%) non-AKI tetanus cases were on ventilator (Table-II).

All tetanus patients received sedatives and muscle relaxants as per PICU protocol. However subsequent renal adjustments were done in the doses in AKI developers. Maximum diazepam requirement was 20 mg/kg/day in one AKI victim who was subsequently discharged. Similarly, maximum magnesium sulphate dose was 100 mg/kg/day q six hourly required by three patients, out of them two were discharged. Phenobarbital was given 5-8 mg/kg/day q 12 hourly and chlorpromazine given 0.5-1mg/kg/day in all the tetanus patients.

Regarding renal replacement therapy (RRT), peritoneal dialysis (PD) was done in four patients with AKI but it did not effect the outcome. Average length of stay was 25 days in AKI cases and 20 days in non AKI cases. (Table-II)

## DISCUSSION

Tetanus is a disease of high mortality ranging 20% to over 50%^11^ Patients usually require prolonged treatment with multiple drugs to control spasm. They are prone to develop various complications, which can alter the course and outcome of the illness.^12^-^15^

Altered renal physiology may be seen in tetanus. Studies have shown that upto 50% of patients with tetanus have a glomerular filtration rate (GFR) lower than 50ml/min in the first or second week of hospitalization.^16^ Acute kidney injury (AKI) is a known complication of tetanus. It has been reported with varied frequency ranging 14% to 39% from different areas. AKI was found to be 31.81% in our case series, reflecting a similar trend.

Studies published so far have used serum creatinine as a marker of AKI in tetanus and so did we. Although serum creatinine is no longer considered as an ideal marker as it does not increase until the GFR has moderately decreased i.e kidney function has already been lost by 25-50%.^17^,^18^

Serum creatinine values are also affected by muscle mass, hydration status, age, sex, gender and method of measurement.^19^ Trends are now toward search for early markers of AKI like serum cystatin C and NGAL (neutrophil gelatinase associated lipocalin.^20^,^21^ AKI was mostly non oliguric (64.29%) in our case series as reported in other previous studies. All the AKI cases belonged to severity grade III and IV of tetanus with the later having the worst outcome (Table-III). This was in contrast to few studies in which acute renal failure was not related to the severity of tetanus.^16^

Association of severity with the development of AKI seems logical because autonomic instability is more marked in severe cases. Renal dysfunction in tetanus is multifactorial and various pathophysiological mechanisms have been suggested.^1^,^9^,^16^ Autonomic instability has been proposed to be the most important factor for the development of AKI, both in adults as well as in paediatric patients.^22^ It was seen in all the AKI developers of our case series.

Uncontrolled muscle spasms causing rhabdomyolysis and ultimately myoglobinuria resulting in acute renal failure has always remained an important factor in adult tetanus patients.^23^ However it has not been emphasized in paediatric population especially in children younger than 10 years as a reason for AKI.^24^,^25^ This may be because children do not have muscle mass to such an extent to cause significant myolysis. In this retrospective review, none was investigated for its development.

Other contributing factors for AKI are the use of nephrotoxic drugs, mechanical ventilation and development of sepsis. Drug related nephrotoxicity was less likely in this series since the creatinine clearance was regularly checked and drugs adjusted accordingly.^26^ Ventilator dependency was seen in 12 (85.71%) AKI patients among whom nine patients (75%) expired, P value 0.04, thus making it a significant risk factor for poor outcome in AKI patients.

The lower the creatinine clearance, the worst was the outcome. Out of 14 AKI victims four had eccl between 75-50% and they were among AKI survivors. Whereas 10 had eccl below 50% i.e stage III according to ‘AKIN’^28^ and nine of them were non survivors, indicating that outcome of AKI is dependent upon its severity also, among other factors.^29^

Sepsis was an important contributor to AKI in this series with CRP being more than 100 in seven cases of AKI, six of them being non-survivors.^30^ Although the difference was large, but due to the small sample size, p value was insignificant (Table-III).

Raised TLC more than 20,000/mm^3^ as a marker of sepsis was seen in 39 (88.63%) cases. Eleven of them developed AKI among which eight expired (72.72%) P value 0.012. (Table-III) Blood Culture positivity was present in 15 cases of tetanus, six of them were AKI cases and five were AKI non-survivors P value-0.001, (Table-III). Only four AKI patients were subjected to RRT (peritoneal dialysis), which did not improve their outcome probably because of late institution.

## CONCLUSION

Development of AKI in tetanus is multifactorial. Major contributors are severity of the tetanus itself, presence of autonomic instability, ventilator dependency, and sepsis. Presence of AKI worsens the outcome of tetanus in terms of survival, length of stay, hospital costs and ventilator days. Need for early rising biomarkers to intervene early is justifiable.
